# Supplementary material for: Efficacy of Low-Level Laser Therapy in a Rabbit Model of Rhinosinusitis
Source: Int J Mol Sci. 2023 Jan 1;24(1):760. doi: 10.3390/ijms24010760 (PMC9820841; doi:10.3390/ijms24010760)
Supplement: Supplementary file 1 [file ijms-24-00760-s001.zip › ijms-2007740-supplementary/Supplementary Table S1.pdf]

Supplementary Table S1. Histopathologic analysis

Groups	Epithelial thickness (μm)	Overall inflammatory cells (cells/mm^2)	Mast cells (cells/mm^2)	Goblet cells (cells/mm^2)
Negative control	13.80±1.05	26.00±1.15	4.67±0.67	92.00±12.86
Positive control	30.05±1.62**	347.20±122.13	29.60±7.78	368.00±16.31**
Natural recovery	27.93±1.01**	307.60±49.59**	30.40±2.56**	334.00±29.57**
Laser-treated	19.53±1.46**	79.20±12.55**	10.40±1.17**	157.20±18.17**

Data are expressed as the mean±S.E. The results were statistically analyzed by Student's *t*-test.

$^{**}P < 0.01$
