# Supplementary figures and images for: Efficacy of Low-Level Laser Therapy in a Rabbit Model of Rhinosinusitis
Source: Int J Mol Sci. 2023 Jan 1;24(1):760. doi: 10.3390/ijms24010760 (PMC9820841; doi:10.3390/ijms24010760)

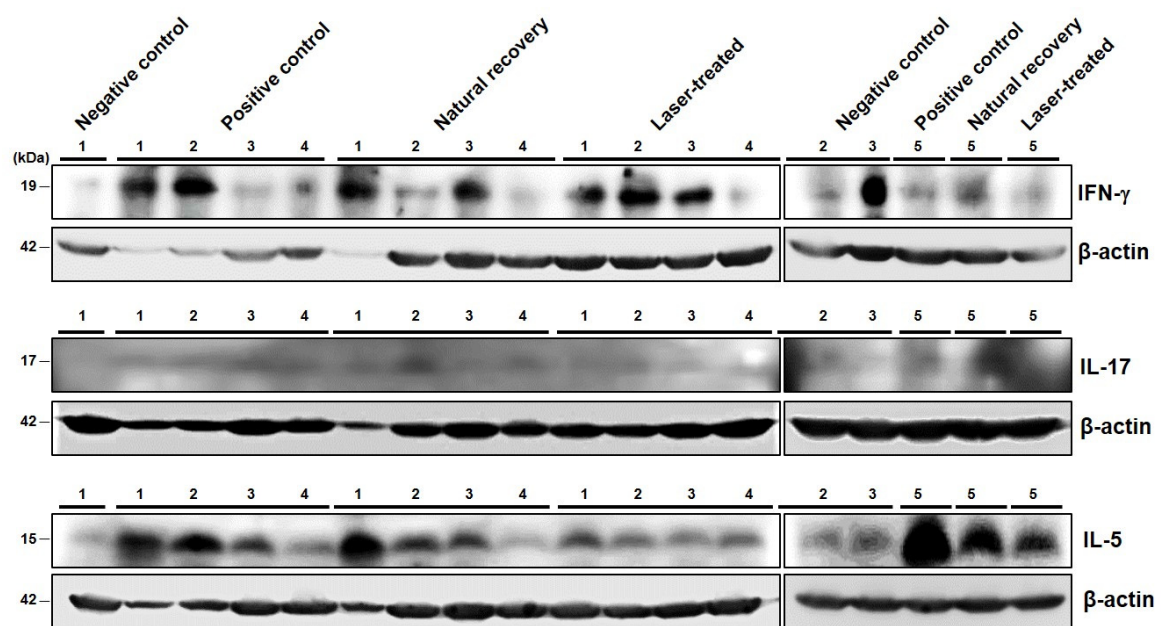

**Figure S1:** Whole Western blotting bands of Figure 3B

Supplement: Supplementary file 1 [file ijms-24-00760-s001.zip › ijms-2007740-supplementary/Supplementary Figure S1.pdf]

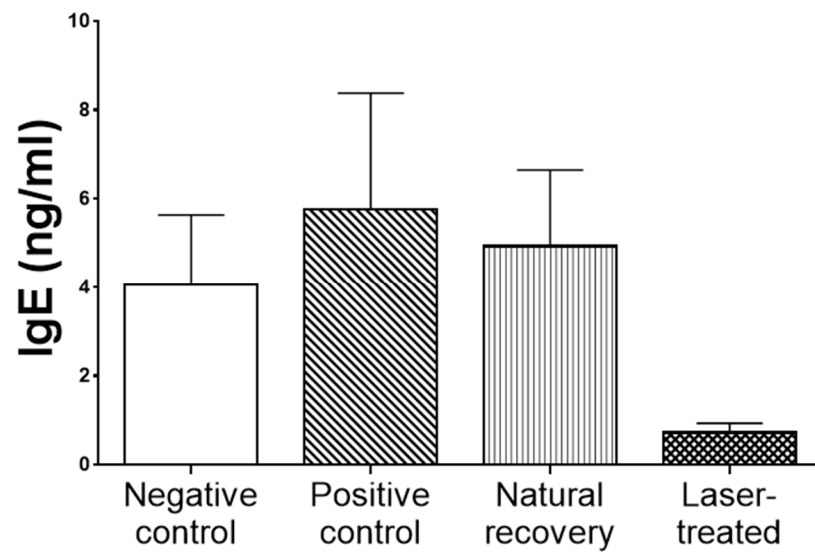

**Figure S2.** Total IgE levels from serum.

Supplement: Supplementary file 1 [file ijms-24-00760-s001.zip › ijms-2007740-supplementary/Supplementary Figure S2.pdf]
